# Skydiving for Beginners: A Journey of Recovery and Hope

**DOI:** 10.1192/pb.bp.114.048371

**Published:** 2015-06

**Authors:** Femi Oyebode

**Figure:**
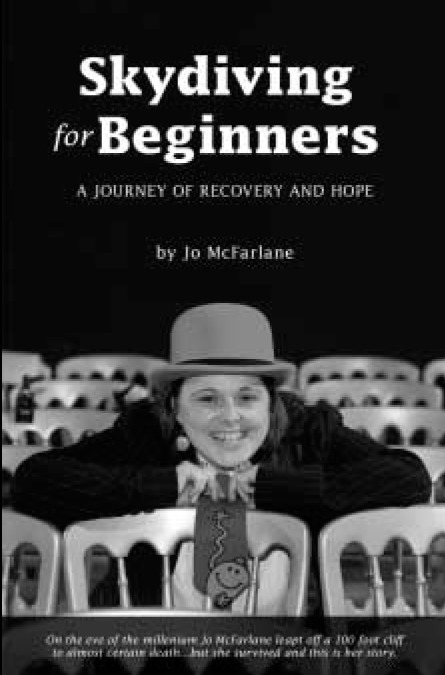


This book will surprise you, shock you, and intrigue you but ultimately it will fill you with admiration and respect for the author. It is the personal memoir of Jo McFarlane, a woman who was born into adversity but who has triumphed, very much against the odds. She is that rare human being: sensitive, thoughtful, positive, driven and without bitterness. A living example of what resilience means in practice.

Her life started in ‘virtual squalor with dry rot and gaping holes in the floor’. She goes on: ‘Ours was a dirty, freezing home infested with vermin’. As if the utter material deprivation was not enough she experienced sexual abuse at the hand of both her father and brother and the circumstances were unspeakable: ‘The politics of my parents’ sex life was played out openly among the children. We knew that he wanted it all the time and that she hated it. This was an enduring source of tension in their marriage and they often embroiled us in the drama [...] One of her avoidance strategies was to have me sleep in their bed between them. I soon became an outlet for his sexual frustration’. The whole family were subjected to her father’s unpredictable moods which ‘like flames could blaze at the slightest provocation and burn for hours; at other times they were extinguished in a breath [...] So unpredictable were his rages that the atmosphere was like a bomb ticking towards its inevitable climax’.

This deprived and abusive childhood formed the backdrop of her psychiatric history in adulthood. Her account of her many admissions, treatments, suicide attempts, and relationships with psychiatrists, nurses and social workers is written with candour. It is an unsparing honesty with which she describes her own behaviour with unswerving clarity and objectivity. There is no sentimentality, self-pity or excuses here. It is an analytic mind that is on display, one that is eloquent and self-assured in how it handles language and ideas. She says of one of her depressive spells: ‘A military metaphor is the most apt I can think of to describe the war zone in my head. It was not a benign melancholy but a splintering of faculties, a torture even to exist. The rapid gunfire of destructive thoughts supplanted my will to survive’. Again, ‘I was so paranoid I thought Kathryn had hidden cameras in my flat, that they were all watching and laughing at my distress, that they could hear what the voices were saying to me and were using them to drive me to suicide. I felt I had to get away from the Royal Edinburgh as far as possible and I boarded a night bus for London. The journey was hell because of my mental state’.

The depiction of life on psychiatric wards, of good relationships with psychiatrists, of the exemplary quality of the interactions with some nurses, and of the kindness and generosity of many people underlines what is admirable and exceptional in mental health services. But, sadly, there are many examples of abuse, of disinterest, of perfunctory interactions, of gross neglect and of errors of judgement. What is impressive is that Jo McFarlane takes the good and the ugly in her stride and she emerges as an astonishing human being.

This memoir stands alongside the great memoirs of Daniel Schreber, Janet Frame, William Styron and Kay Redfield Jamison. It sheds light on the intersections of disrupted attachment in early life, of traumatising abuse and of biological vulnerability to psychosis. It reveals the unheard but real voice of a fragile self that is masked by serious illness. And McFarlane’s own ambition in writing this book is to be ‘an invitation to others, through encouragement and example, to embrace their talents with pride and joy’. I think she has succeeded marvellously.

